# Accuracy of intraoral scan images in full arch with orthodontic brackets: a retrospective in vivo study

**DOI:** 10.1007/s00784-021-03792-0

**Published:** 2021-01-21

**Authors:** Young-Kyun Kim, So-Hyun Kim, Tae-Hyun Choi, Edwin H. Yen, Bingshuang Zou, Yonsoo Shin, Nam-Ki Lee

**Affiliations:** 1 Department of Oral and Maxillofacial Surgery, Seoul National University Bundang Hospital, Seongnam, South Korea; 2 Department of Dentistry & Dental Research Institute, School of Dentistry, Seoul National University, Seoul, South Korea; 3 Department of Orthodontics, Seoul National University Bundang Hospital, Seongnam, South Korea; 4 Department of Orthodontics, Faculty of Dentistry, University of British Columbia, Vancouver, British Columbia Canada

**Keywords:** Accuracy, Intraoral scan images, Orthodontic brackets, Digital impression

## Abstract

**Objectives:**

The purpose of this retrospective study was to evaluate the accuracy of intraoral scan (IOS) images in the maxillary and mandibular arches with orthodontic brackets.

**Material and methods:**

From digital impressions of 140 patients who underwent orthodontic treatment, consecutive IOS images were selected based on standardized inclusion criteria: Two pre-orthodontic IOS images (IOS1 and IOS2) of permanent dentition with fully erupted second molars and IOS images obtained immediately after orthodontic bracket bonding (IOSb). Superimpositions were performed to evaluate the reproducibility of repeated IOS images. Accuracy of IOSb images was analyzed by comparing the average surface errors between IOS1c and IOS2c images, which were IOS images cut based on the same region of the interest as between IOS1 and IOSb images.

**Results:**

A total of 84 IOS images was analyzed. The average surface errors between IOS1 and IOS2 images were 57 ± 8 μm and 59 ± 14 μm in the maxillary and mandibular arch, respectively, and their reliability was almost perfect. The average errors between IOSb and IOS1c images exhibited an increase, which measured 97 ± 28 μm in the maxillary arch and 95 ± 29 μm in the mandibular arch. These surface deviations between IOSb and IOS1c images were significantly larger in each region as well as entire dentition (*P* < 0.001) compared to those between IOS1c and IOS2c images.

**Conclusions:**

The average surface errors of the scans with brackets showed increased values compared with those without brackets. This suggests that orthodontic brackets could affect the trueness of intraoral scan images.

**Clinical relevance:**

It is necessary for clinicians to consider the effect of brackets on digital impression when using IOS images in orthodontic patients.

## Introduction

Digital technologies have been applied to obtain digital models, calculate spacing and crowding, simulate virtual tooth movements, virtualize orthognathic surgery, and fabricate surgical wafers [1–3]. Measurements on 3-dimensional (3D) dental models, which were rendered from either CBCT or extraorally digitized plaster casts, were clinically reliable and accurate compared to those from current gold standard plaster models [4, 5]. Furthermore, 3D printing of some orthodontic appliances has been introduced [6, 7].

Intraoral scanning is efficient, less stressful, and exhibits good acceptability [8–12], which allows clinicians to obtain patients’ occlusion digitally without taking conventional impressions. Recently, some studies reported that the accuracy and reproducibility of intraoral scan images varies depending on different types of scanners [13–15]. Jeong et al. demonstrated a precision range from 58 μm to 116 μm with two types of intraoral scanners [16]. Kim et al. found that, during scanning complete-arch models with different cavity preparations, the Trios scanner showed the highest precision (34.7 μm), and the E4D scanner the lowest precision (357.1 μm) [17]. However, most of the studies were performed by scanning plaster or custom reference models, not directly from intraoral conditions of patients [13, 18, 19].

It is challenging to obtain accurate intraoral scan (IOS) images of full arches with various fixed orthodontic appliances, under intraoral environments with saliva, restorations, debridement, tongue, and cheeks. Considering these clinical conditions, further studies on intraoral scanning are needed. To the authors’ knowledge, there have been few studies on the accuracy of intraoral digital impressions of full arches with orthodontic appliances. Prior to such studies, we evaluated the reproducibility of IOS images acquired in maxillary and mandibular arches as baseline and then the accuracy of those with brackets. In particular, this study tested the hypothesis that among digital impressions, the accuracy of IOS images from dentitions with bonded brackets would not differ from those without brackets. The specific purpose of this study was to evaluate the trueness of IOS images with orthodontic brackets in the maxillary and mandibular arches.

## Material and methods

**Fig. 1:**
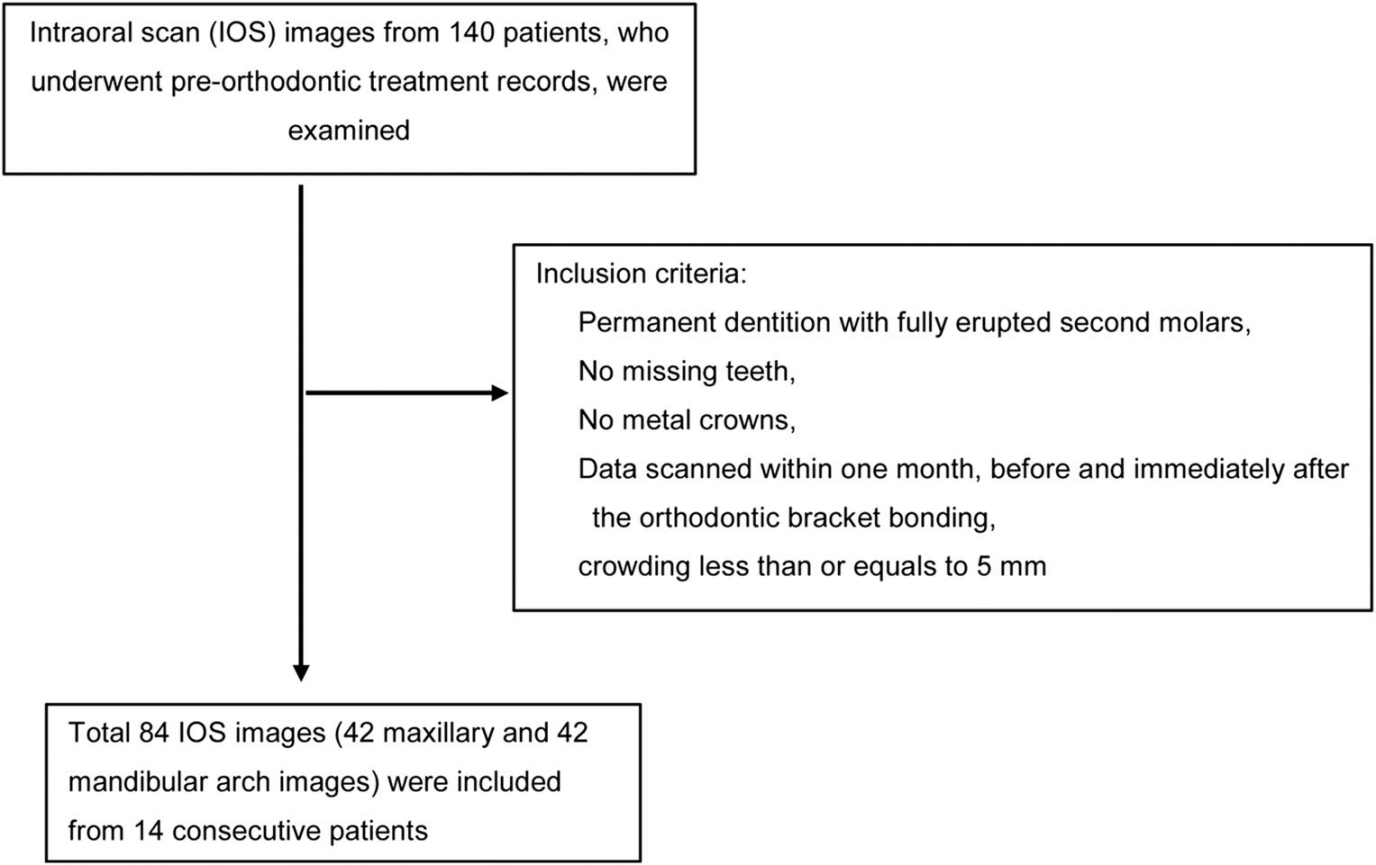
Cohort diagram showing selection of intraoral scan images

This retrospective study was reviewed and approved by the Institutional Review Board of Seoul National University Bundang Hospital (B-1911/574-105). Digital impressions were collected from 140 patients who underwent pre-orthodontic treatment records from January 2017 to January 2020 at Seoul National University Bundang Hospital. The IOS images were selected by the following inclusion criteria: (1) permanent dentition with fully erupted second molars, (2) no missing teeth, (3) no metal crowns, and (4) intraoral scan data within 1 month, before and immediately after the orthodontic bracket bonding. The IOS images with crowding greater than 5 mm were excluded (Fig. 1).

### Image acquisition

All IOS images were obtained by an experienced orthodontist (SH. K), who was specially trained and had performed dozens of practice scans, using an intraoral scanner (Carestream CS 3600®, Carestream Dental, Atlanta, GA) according to the manufacturer’s instructions. The scanning procedure was performed for each patient in the dental chair in an upright position [8, 11]. The pre-orthodontic digital impressions acquired twice with an interval of 2 weeks were regarded as the first and second IOS images (IOS1 and IOS2, respectively). Digital impressions obtained immediately after bracket bonding without archwire insertion were defined as IOS images with orthodontic brackets (IOSb). Each IOS dataset was exported as Standard Tessellation Language files with the CS imaging software (Carestream Dental, Atlanta, GA) and was generated as a digital model for analysis.

### Superimposition and data acquisition

**Fig. 2:**
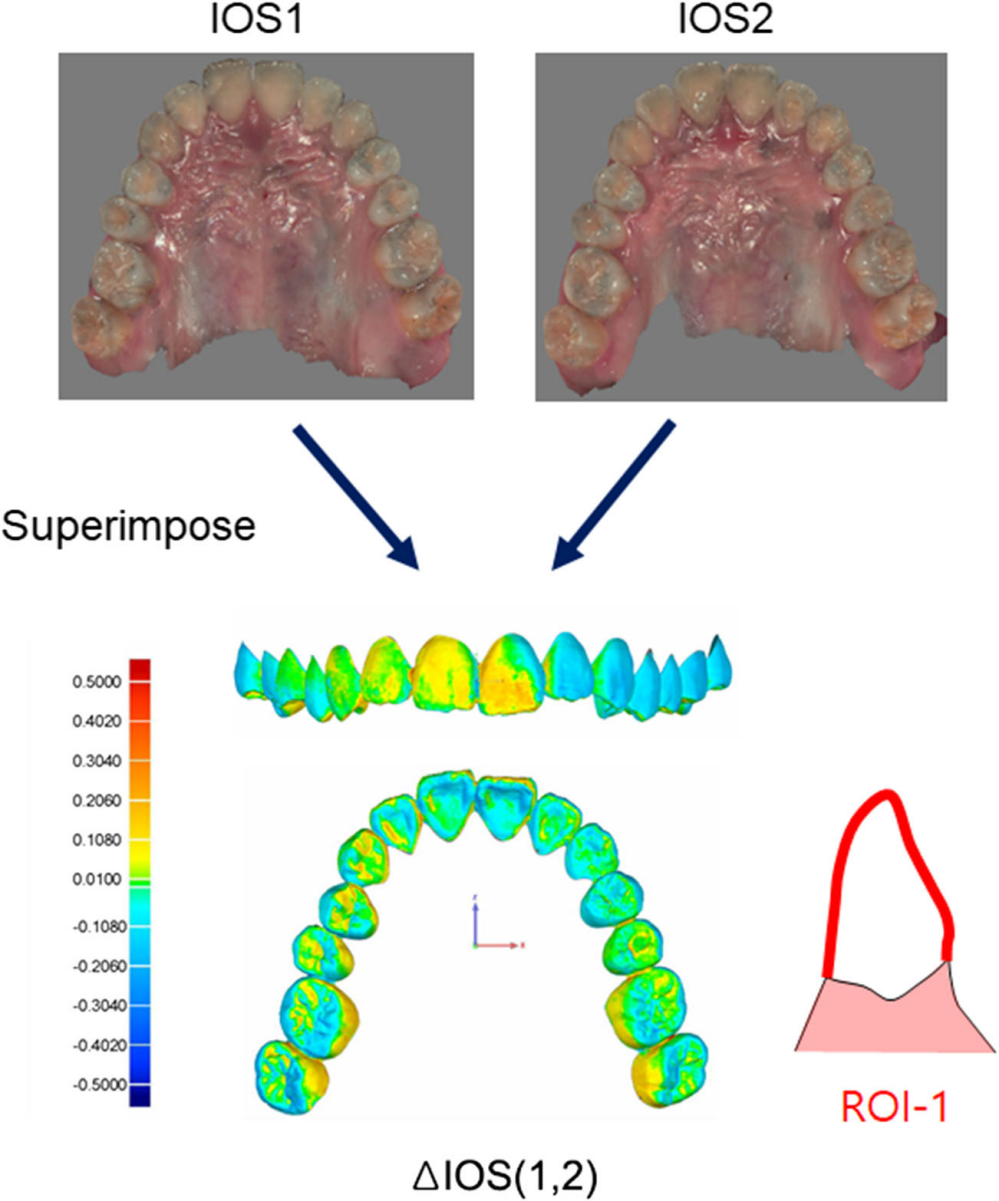
Study workflow of intraoral scan (IOS) images taking, their superimposition, and analysis using the Geomagic software program. To evaluate the reproducibility of IOS images, IOS1 and IOS2 images scanned twice at 2-week intervals, were superimposed, and average surface errors between them [Δ IOS (1, 2)] were measured based on the first region of interest (ROI-1), which were established as the bucco-lingual surfaces of the entire teeth above the gingival line

**Fig. 3:**
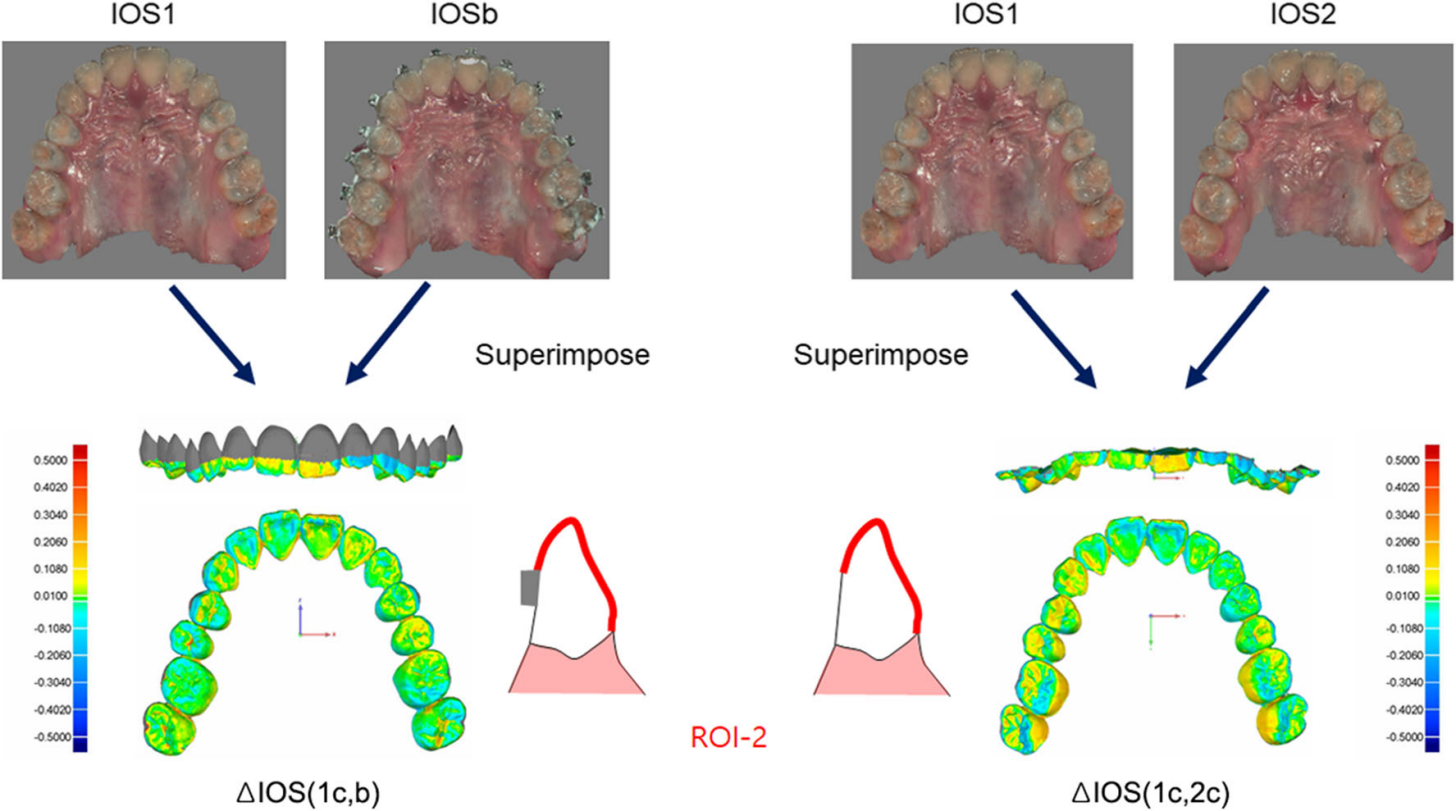
Study workflow of intraoral scan (IOS) images taking, their superimposition, and analysis using the Geomagic software program. To assess trueness of the IOS after bracket bonding (IOSb) images, IOS1 and IOSb images were superimposed, and average surface errors [Δ IOS (1c, b)] were obtained based on the second ROI (ROI-2), which was the occluso-buccal surfaces of the teeth above the upper-most margin of the bracket bases and the lingual surfaces of the teeth. Also, the superimposed IOS1 and IOS2 images were cut according to ROI-2 and defined as IOSc images (IOS1c and IOS2c, respectively) for the comparison of the surface errors [Δ IOS (1c, 2c)]

The 3-dimensional analysis software (Geomagic Qualify 2013®, 3D-systems, Morrisville, NC) was used to superimpose the IOS images with the best-fit algorithm. The first region of interest (ROI-1) used to evaluate reproducibility was the bucco-lingual surfaces of the entire teeth above the gingival line in the IOS1 and IOS2 images (Fig. 2). The second ROI (ROI-2) comprised the occluso-buccal surfaces of the teeth above the upper-most margin of the bracket bases and the lingual surfaces of the teeth (Fig. 3). To assess the trueness of IOSb images, superimposed IOS1 and 2 images were cut according to ROI-2 and defined as IOSc images (IOS1c and IOS2c, respectively; Fig. 3). Differences between the IOS images were presented as average surface errors of the total arch, anterior, premolar, and molar regions in the maxillary and mandibular dentition.

### Statistical analysis

Power analysis with *α* = 0.05, effect size = 0.90, and power (1-β) = 0.80 showed requirement of 13 surface error values (G* Power 3.1.9.7;Heinrich-Heine-University, Dϋsseldorf, Germany) [20]. Accordingly, more than 26 IOS images were needed because one average surface error was derived from a pair of IOS images.

When evaluating the accuracy of digital images, reproducibility is usually defined as the agreement among repeated data and trueness as closeness to the reference data [9, 18]. To evaluate the reproducibility of IOS images as baseline, the average surface errors, the image differences obtained after superimpositions of IOS1 and IOS2, were determined via descriptive statistics and intraclass correlation coefficients (ICC). Conformity, how well the average surface errors among IOS1c, IOS2c, and IOSb images accurately correspond to each other, was analyzed by Lin’s concordance correlation coefficients (CCC) and Bland-Altman analysis. In addition, the trueness of IOSb images was analyzed by comparing the average errors between IOSb and IOS1c and those between IOS1c and IOS2c using the Mann-Whitney *U* test. To identify the difference between the average surface image errors of maxillary and mandibular arches, the Mann-Whitney *U* test was used. The Kruskal-Wallis test was performed to evaluate surface errors among the anterior, premolar, and molar regions in both arches. All statistical data were analyzed with the SPSS software (Version 22.0, IBM, Armonk, NY).

## Results

A total of 84 IOS images, consisted of 42 from maxillary and 42 from mandibular arches, was selected according to the inclusion criteria (Table 1, Fig. 1). According to the post hoc power analyses with *α* (two-tailed) = 0.05, effect size = 0.9, and *n* = 14, the estimated power (1−β) of the study was 0.86.

**Table 1:** Characteristics of IOS1, IOS2, and IOSb images

	IOS images		
Dentition	IOS1	IOS2	IOSb
Maxilla (*n*, 42)	14	14	14
Mandible (*n*, 42)	14	14	14

*IOS1* first intraoral scan as pre-orthodontic digital impression; *IOS2* second intraoral scan before bracket bonding; *IOSb* IOS with orthodontic brackets after bonding

### Average surface errors [△ IOS (1, 2)] and their reliability between repeated IOS images in maxillary and mandibular dentition

As presented in Table 2, the average surface error in the total arch between the IOS1 and IOS2 images was 57 ± 8 μm in the maxillary dentition and 59 ± 14 μm in the mandibular dentition. There were no significant differences of the average errors among anterior, premolar, and molar regions in the maxillary and mandibular arches. In addition, intra-observer reliability of average surface errors was almost perfect in the total and anterior regions of IOS1 and IOS2 images (0.911 ≤ ICC ≤ 0.991) and moderate in the maxillary premolar and molar regions (0.435 ≤ ICC ≤ 0.447).

**Table 2:** Average surface errors and their reliability between repeated IOS images

Dentition	Region	Average surface errors [Δ IOS (1, 2); unit, μm]							
		Mean	SD	Max	Min	95% CI		P^*^	ICC [95% CI]
						Lower	Upper		
Maxilla	Total	57	8	77	48	53	62	n/a	0.991 [0.934/0.999]
	Anterior	54	11	72	33	48	60	0.201	0.944 [0.580/0.994]
	Premolar	54	12	71	18	47	61		0.447 [−1.715/0.936]
	Molar	63	13	86	46	56	71		0.435 [−4.091/0.941]
Mandible	Total	59	14	82	35	51	67	n/a	0.907 [0.290/0.990]
	Anterior	55	14	81	38	47	63	0.262	0.983 [0.831/0.998]
	Premolar	48	11	70	31	42	54		0.914 [0.373/0.991]
	Molar	61	21	97	33	49	73		0.911 [−0.013/0.991]

Δ *IOS (1, 2)* the average surface errors, representing image difference obtained after superimposition and cutting of ISO1 and IOS2 images according to ROI-1; *IOS1* first intraoral scan as pre-orthodontic digital impression;* IOS2* second intraoral scan before bracket bonding; *Total* whole dentition; anterior, premolar, and molar regions were divided by contact points in the dentition after superimposition of the two images; *Max* maximum; *Min* minimum; *CI* confidence interval

^*^Kruskal Wallis test; n/a; not applicable; ICC, intraclass correlation coefficients; ICC > 0.8/0.6/0.4/0.2 represent almost perfect, substantial, moderate, or mediocre reliability, respectively

### Reliability and conformity of average surface errors among IOS1c, IOS2c, and IOSb images in maxillary and mandibular dentition

Intra-observer reliability of average surface errors was almost perfect between IOS1c and IOS2c [Δ IOS (1c, 2c)] images (0.989 ≤ ICC ≤ 1.000; Table 3).

**Table 3:** Reliability and conformity of the average surface errors among IOS1c, IOS2c, and IOSb images

		Reliability	Conformity		
		ICC [95% CI]		CCC [95% CI]	
		Δ IOS (1c, 2c)	Δ IOS (1c, b)	Δ IOS (2c, b)	Δ IOS (1c, 2c)
Maxilla	Total	1.000 [0.997/1.000]	0.013 [−0.457/0.478]	0.014 [−0.458/0.479]	0.999 [0.994/1.000]
	Anterior	0.999 [0.991/1.000]	0.307 [−0.662/0.892]	0.331 [−0.651/−0.535]	0.997 [0.975/1.000]
	Premolar	0.999 [0.996/1.000]	−0.342 [−0.882/0.586]	−0.330 [−0.877/0.591]	0.999 [0.990/1.000]
	Molar	0.995 [0.889/0.999]	−0.033 [−0.502/0.451]	−0.045 [−0.550/0.484]	0.987 [0.948/0.997]
Mandible	Total	0.994 [0.878/0.999]	−0.641 [−0.894/−0.076]	−0.577 [−0.867/0.004]	0.984 [0.923/0.997]
	Anterior	1.000 [0.998/1.000]	0.538 [0.198/0.762]	0.550 [0.207/0.773]	1.000 [0.999/1.000]
	Premolar	1.000 [1.000/1.000]	0.600 [−0.115/0.905]	0.604 [−0.102/0.906]	1.000 [0.999/1.000]
	Molar	0.989 [0.906/0.999]	−0.476 [−0.916/0.484]	−0.607 [−0.947/0.372]	0.972 [0.785/0.843]

*Δ IOS (1c, 2c) [or Δ IOS (1c, b), or Δ IOS (2c, b)] *the average surface errors, representing image difference obtained after superimposition and cutting of the two IOS images according to ROI-2; *ICC* intraclass correlation coefficients; ICC > 0.8/0.6/0.4/0.2 represent almost perfect, substantial, moderate, or mediocre reliability, respectively; *CCC* Lin’s concordance correlation coefficients; CCC of > 0.8/0.6/0.4/0.2 or ≤ 0.2 represent almost perfect, substantial, moderate, mediocre, or low conformity, respectively; *CI* confidence interval

**Fig. 4:**
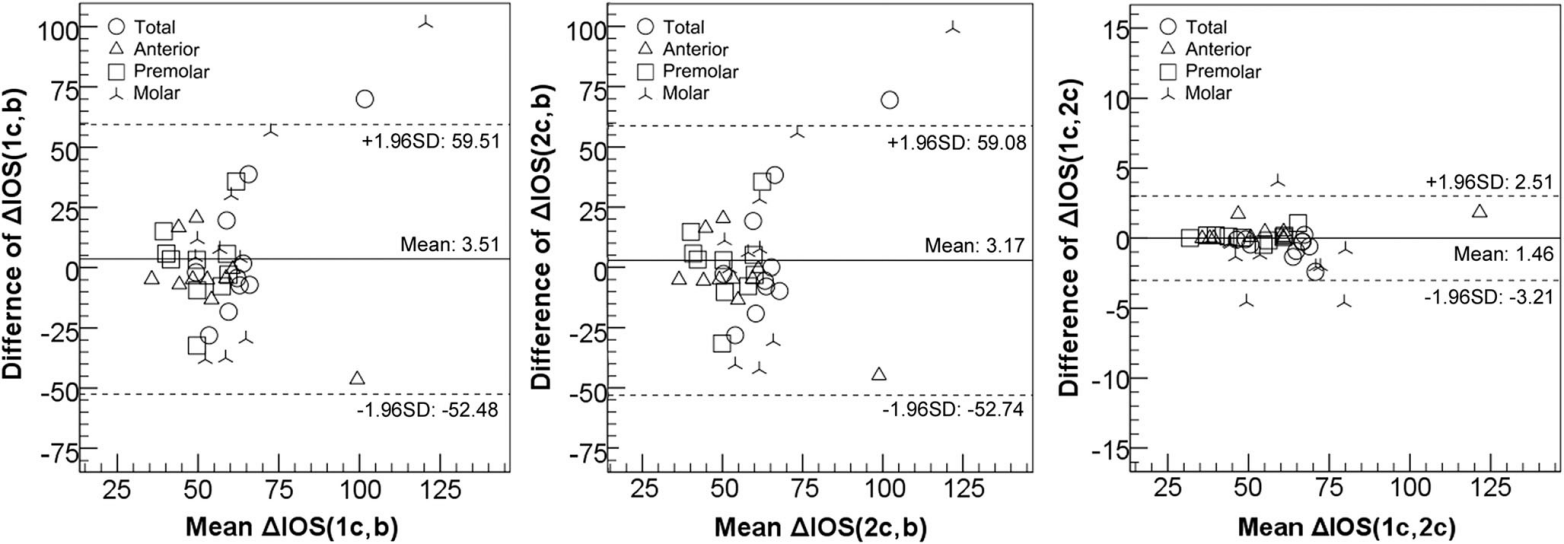
Bland-Altman plot of average surface errors among IOS1c, IOS2c, and IOSb images

In conformity, all regions presented high values of CCC (≥ 0.984) in the average surface errors between IOS1c and IOS2c images. However, the regions presented low to moderate values of CCC in the average surface errors between IOS1c (or 2c) and IOSb images [Δ IOS (1c, b) and Δ IOS (2c, b); Table 3]. In addition, Bland-Altman plot showed larger deviations in Δ IOS (1c, b) and Δ IOS (2c, b) compared to those of Δ IOS (1c, 2c) (Fig. 4).

### Average surface errors among IOS1c, IOS2c and IOSb images, and their comparisons in the maxillary/mandibular dentition

As shown in Table 4, the average errors between IOSb and IOS1c images were significantly larger in the total, anterior, premolar, and molar regions in the maxillary arch compared to those between IOS1c and IOS2c images (all *P* < 0.001). Likewise, in the mandibular arch, the average errors between IOSb and IOS1c images exhibited significantly larger values in all regions (all, *P* < 0.001). There were no significant differences of average errors among the regions (anterior, premolar, and molar) in either the maxillary or mandibular arch.

**Table 4:** Average surface errors among IOS1c, IOS2c, and IOSb images and their comparisons in the maxillary/mandibular dentition

Dentition	Region	Average surface errors [Δ IOS (1c, 2c); unit, μm)							Average surface errors [Δ IOS (1c, b); unit, μm]							
		Mean	SD	Max	Min	95% CI		P^*^	Mean	SD	Max	Min	95% CI		P^*^	*P*^*†*^
						Lower	Upper						Lower	Upper		
Maxilla	Total	55	10	69	40	49	60	n/a	97	28	150	57	81	112	n/a	< 0.001
	Anterior	45	9	61	34	39	50	0.274	93	40	209	44	70	117	0.944	< 0.001
	Premolar	47	9	66	37	42	53		96	36	157	38	75	117		< 0.001
	Molar	53	14	77	38	45	61		91	39	159	10	68	113		< 0.001
Mandible	Total	58	13	82	42	51	66	n/a	95	29	159	53	78	112	n/a	< 0.001
	Anterior	54	24	122	36	40	68	0.310	94	27	132	32	78	109	0.805	< 0.001
	Premolar	45	12	74	31	38	52		101	63	294	35	65	138		< 0.001
	Molar	54	17	83	33	44	64		95	27	144	59	79	110		< 0.001

*Δ IOS (1c, 2c) [or Δ IOS (1c, b)] *the average surface errors, representing image difference obtained after superimposition and cutting of the two IOS images according to ROI-2; *Max* maximum; *Min* minimum; *CI* confidence interval

^*^Kruskal Wallis test

^†^Mann Whitney *U* test; *n/a* not applicable

### Average surface errors of repeated IOSc or IOSb and IOS1c images between maxillary and mandibular arches

Average surface errors of IOSb and IOS1c images showed larger values compared with those of repeated IOSc images, but not significantly different among the regions between the maxillary and mandibular arches (Table 5).

**Table 5:** Average surface errors of IOS1c and 2c or IOSb and 1c images between maxillary and mandibular arches

Region	Average surface errors [Δ IOS (1c, 2c); unit, μm]					Average surface errors [Δ IOS (1c, b); unit, μm]				
	Maxilla		Mandible		*P*^*†*^	Maxilla		Mandible		*P*^*†*^
	Mean	SD	Mean	SD		Mean	SD	Mean	SD	
Total	55	10	58	13	0.635	97	28	95	29	0.701
Anterior	45	9	54	24	0.352	93	40	94	27	0.401
Premolar	47	9	45	12	0.285	96	36	101	63	0.839
Molar	53	14	54	17	0.946	91	39	95	27	0.874

*Δ IOS (1c, 2c) [or Δ IOS (1c, b)] *the average surface errors, representing image difference obtained after superimposition and cutting of the two IOS images according to ROI-2

^†^Mann Whitney *U* test

## Discussion

With increasing needs for digital impressions, intraoral scanning for orthodontic treatment as well as diagnosis may be necessary under conditions of orthodontic appliances such as brackets. For this reason, the accuracy of IOS images on dental arches with orthodontic appliances needs to be evaluated. This study sought to compare the accuracy between IOS images obtained with and without brackets. As a baseline, we evaluated the reproducibility of IOS images acquired directly from both arches. The specific aim of the study was to evaluate the trueness of IOS images with orthodontic brackets.

Regarding the reproducibility of digital images in this study, the ICC of average surface errors between IOS1 and IOS2 images was almost perfect in the total regions, which implied that intraoral scanning was reliable (Table 2). Consistent with this result, Kirschneck et al. reported that intraoral scanning showed sufficient reliability and validity for clinical use, with higher deviations in intraoral scan images compared to extraoral scan of plaster models [21].

The difference of IOS1 and IOS2 images showed mean surface deviations ranging from 57 μm to 59 μm in the total arch of the maxillary and mandibular dentitions. A recent study on digital impressions using CS 3600 demonstrated a precision of 46 μm ± 5 μm, but they evaluated it on the maxillary plaster models [22]. A systemic review on the accuracy of scanning reported that the deviation values of the IOS image of the entire dentition by intraoral and laboratorial scanners were between 17 μm and 378 μm and suggested that intraoral scanning of complete dentition exhibited adequate accuracy [15]. In addition, Lim et al. reported a different range of deviation according to type of intraoral scanner, with the Trios showing greater precision (mean error, 52.3 μm) compared to iTero (60.5 μm) in 10 repeated patient oral cavity scans [23]. Some studies found that image errors seemed to result from various factors such as type of scanner, performer’s experience, existence of crowding, and scanning technique [23–26]. In our study, the surface error range of repeated IOS images is similar to or slightly larger than those of previous studies. This difference can be partially explained due to the scanner types, which have different data acquisition methods and light sources [17]. It can also be attributed to in vivo scanning, which exhibited lower accuracy than ex vivo scanning due to its weakness such as patient movement, limited access in intraoral space, and saliva [27].

To evaluate the effect of orthodontic brackets on surface errors of acquired IOS images, we superimposed the IOSb and IOS1 images and the IOS1 and IOS2 images and cut them according to ROI-2 for comparison of surface errors. The ROI-2 was established as the lingual and occluso-buccal surfaces of teeth above the upper-most margin of the bracket bases for the following reasons: (1) the accuracy of these areas could be closely associated with fabrication of additional orthodontic appliances or printing surgical wafers; (2) the cervical surfaces of the teeth under the brackets may be affected by the residual bonding resin; (3) the technical difficulty of exact exclusion of the bracket area after superimposition.

Regarding the reliability and conformity of the average surface errors among IOS1c, IOS2c, and IOSb images, the surface errors between IOS1c and IOS2c images (without brackets) were reliable and almost identical. On the other hand, low to moderate concordance of the average surface errors between IOS1c (or 2c) and IOSb images (with brackets) indicates larger deviations compared to those between IOS1c and IOS2c images (Table 3 and Fig. 4).

The difference of IOSb and IOS1c images exhibited an increase in average surface errors of 97 μm in the maxillary arch to 95 μm in the mandibular arch. These deviations between IOSb and IOS1c images were significantly larger in each region as well as the entire dentition compared to those between IOS1c and IOS2c images (Table 4). This means that IOSb images have lower trueness compared to IOSc images, and suggests that orthodontic brackets may affect the accuracy of IOS images. Therefore, the null hypothesis was rejected. This increase of surface error may be attributed to the following reasons: (1) repeated scanning of the bracket area after bonding and (2) scattered reflection resulting from scanning of metal or ceramic brackets. Park et al. showed that a digital image with lingual brackets had less accuracy than that of buccal brackets due to the short interbracket distance and difficulty with scanning [28]. However, they measured 2-dimensional inter-molar widths in ex-vivo scans of ideal dental models with brackets.

Regarding clinical application, a recent study on the precision of 3D-printed splint generated from dental models with different offsets reported that 100–200 μm clearance was required for adequate adaptation of the splints [29]. Another validation study of 3D-printed wafers for orthognathic surgery found clinically acceptable mean distance error of 400 μm [1]. The surface errors of 95 to 97 μm (max, 150 to 159 μm) after bracket bonding in this study imply that intraoral scanning can be used to fabricate removable orthodontic appliances. To the best of our knowledge, there has been no information on the maximum acceptable errors of IOS images to be accurate enough for clinical use, and further study is needed.

In relation to regional differences, there was no significant difference of mean errors among anterior, premolar, and molar regions of repeated IOS images in each arch (Table 2). Differences in the regional errors of IOS images between the maxillary and mandibular arches were not found, irrespective of orthodontic brackets (Table 5). Lim et al. reported that scan images between maxillary and mandibular arches presented no significant difference [23]. On the other hand, Flügge et al. demonstrated larger average deviation (57 μm) in the maxilla compared to the mandible (43 μm) in full arch scans [27]. They reported that image errors resulted from steep-angled maxillary anterior teeth and complex anatomical shape of the molar area. In addition, another in vitro study found over−/under-estimated errors in the anterior and posterior area according to various scanners [19], adopting single image- or video-based system [30]. The difference in our results might be attributed to the specific ROI-2 based on bracket base, and partially explained by video-based scan system, which reported to be less influenced by the region being scanned [23].

The limitations of this study could involve the small sample size of IOS images, use of different brackets, and restriction of ROI. Further studies are needed to evaluate the influence of the amount of crowding, materials, and size of brackets on the accuracy of IOS images in larger samples. Moreover, it may be necessary to examine whether the buccal and lingual positions of brackets will have different effects on the accuracy of IOS images.

## Conclusions

The average surface errors of the scans with brackets showed increased values compared with those without brackets. This suggests that orthodontic brackets could affect the trueness of intraoral scan images.
